# Integrating biocomputational techniques for vaccine development for glioblastoma multiforme: a possible way of enhancing precision

**DOI:** 10.3389/fimmu.2026.1835999

**Published:** 2026-05-07

**Authors:** Kehinde Alare, Tope Odunitan, Taiwo Alare, Tirenioluwa Ojo, Folasade Alare, Uthman Uthman

**Affiliations:** 1 Department of Medicine, Ladoke Akintola University of Technology, Ogbomoso, Nigeria; 2 Department of Biochemistry, Ladoke Akintola University of Technology, Ogbomoso, Nigeria; 3 Department of Mechanical Engineering, Stony Brook University, New York, NY, United States; 4 Critical Care Department, Virginia Commonwealth University, Richmond, VA, United States; 5 Department of Neurosurgery, National Hospital Abuja, Abuja, Nigeria

**Keywords:** biocomputational, glioblastoma multiforme, immunotherapy, personalized, vaccine

## Abstract

Glioblastoma multiforme (GBM) is the most aggressive primary brain tumor in adults, with poor survival despite multimodal therapy. Tumor heterogeneity, immune evasion, and recurrence limit the effectiveness of current treatments, necessitating novel therapeutic strategies. Vaccine-based immunotherapy aims to induce tumor-specific immune responses but has been challenged by antigen variability and an immunosuppressive tumor microenvironment. Biocomputational techniques have transformed vaccine development by enabling precise identification of immunogenic epitopes and neoantigens. Methods such as reverse vaccinology, immunoinformatics, and artificial intelligence facilitate the rational design of multi-epitope and personalized vaccines. Integration of multi-omics data further enhances target selection and therapeutic precision. Despite these advances, challenges including limited predictive accuracy and translational barriers persist. Overall, biocomputationally driven vaccine design offers a promising pathway toward precision immunotherapy and improved outcomes in GBM.

## Introduction

Glioblastoma multiforme (GBM) is the most aggressive and lethal primary brain tumor in adults, characterized by rapid proliferation, diffuse infiltration, and profound molecular heterogeneity (1, 2). Despite advances in neurosurgical techniques, radiotherapy, and chemotherapy, the prognosis remains poor, with median survival rarely exceeding 15–18 months following diagnosis (3–5). The current standard of care is maximal safe surgical resection followed by radiotherapy and temozolomide which offers only modest survival benefit, largely due to intrinsic tumor resistance, recurrence, and the presence of a highly immunosuppressive tumor microenvironment (6, 7). These challenges underscore the urgent need for innovative therapeutic strategies capable of achieving durable tumor control.

A promising immunotherapeutic strategy for promoting tumor-specific immune responses against cancerous cells is the use of cancer vaccines (8). Vaccine approaches for GBM have focused on patient-specific neoantigens resulting from somatic mutations as well as tumor-associated antigens like EGFRvIII (9, 10). However, tumor heterogeneity, antigen escape, and host immune response variability have hindered the success of traditional vaccine development. More accurate and customized vaccine design strategies are required due to the intricacy of GBM biology (11–13).

Recent advances in biocomputational techniques, including immunoinformatics, reverse vaccinology, structural bioinformatics, machine learning, and multi-omics integration have transformed the landscape of vaccine development (14, 15). These tools enable high-throughput identification of immunogenic epitopes, prediction of major histocompatibility complex (MHC) binding affinity, assessment of antigenicity and population coverage, and in silico modeling of immune responses (16). By integrating genomic, transcriptomic, and proteomic data, computational platforms facilitate the rational design of personalized, multi-epitope vaccines tailored to the unique molecular profile of each tumor (17–19).

This review explores the integration of biocomputational methodologies into GBM vaccine development, highlighting current advances, translational challenges, and future opportunities for precision neuro-oncologic immunotherapy.

## Methods

### Study design

This review was conducted as a comprehensive narrative synthesis with elements of systematic literature appraisal to evaluate the role of biocomputational techniques in vaccine development for Glioblastoma multiforme (GBM). The methodology was designed to ensure transparency, reproducibility, and broad coverage of relevant interdisciplinary studies spanning oncology, immunology, and computational biology.

### Search strategy

A structured literature search was performed across multiple electronic databases, including PubMed/MEDLINE, Scopus, Web of Science, and Embase, from database inception to February 2026. The search strategy combined MeSH terms and free-text keywords. Core search terms included:

“glioblastoma” OR “GBM” AND “vaccine” OR “cancer vaccine” OR “immunotherapy” AND “neoantigen” OR “tumor antigen” AND “immunoinformatics” OR “computational vaccinology” OR “reverse vaccinology” OR “machine learning” OR “artificial intelligence”.

Boolean operators (AND/OR) and truncation were applied to optimize sensitivity and specificity. Reference lists of included articles and relevant reviews were manually screened to identify additional studies.

### Eligibility criteria

Studies were included if they:

- Focused on GBM or central nervous system tumors with translational relevance to GBM
- Investigated vaccine-based immunotherapy (peptide, dendritic cell, nucleic acid, or neoantigen vaccines)
- Incorporated computational, bioinformatic, or multi-omics approaches
- Were published in peer-reviewed journals in English

Exclusion criteria included:

- Non-English publications
- Conference abstracts without full data
- Studies lacking relevance to vaccine development or computational methods

### Study selection

All retrieved records were independently screened based on titles and abstracts, followed by a full-text review for eligibility. Before screening, duplicates were taken out. Disagreements about which studies to include were worked out through discussion and agreement.

### Data extraction and synthesis

Relevant data were extracted, including study design, computational tools utilized, vaccine platform, target antigens, and principal findings. The extracted data were qualitatively synthesized, focusing on the identification of trends, methodological strengths, limitations, and translational relevance.

### Critical appraisal

Included studies were critically evaluated for methodological rigor, including study design, sample size, validation approaches, and reproducibility of computational methods. Particular attention was given to discrepancies between in silico predictions and clinical outcomes.

### Reporting

This review was done following standard rules for combining evidence, and it is said to make the results of computational vaccine development for GBM clearer, easier to reproduce, and more open to critical interpretation.

## Molecular and immunological landscape of GBM

The highly immunosuppressive tumor microenvironment and significant molecular heterogeneity of glioblastoma multiforme (GBM) make treatment intervention and vaccine development challenging (20, 21). At the genomic level, GBM shows a variety of changes related to oncogenic signaling pathways, including TP53 mutations, PTEN loss, EGFR amplification and mutation (including EGFRvIII), and dysregulation of the PI3K/AKT/mTOR axis. GBM is further classified into biologically distinct subtypes based on the status of isocitrate dehydrogenase (IDH) mutations, with IDH-wildtype tumors typically having a worse prognosis (22–26). Furthermore, responsiveness to alkylating chemotherapy is influenced by O6-methylguanine-DNA methyltransferase (MGMT) promoter methylation, illustrating the intricate relationship between genetics and therapeutic results. Uniform vaccine targeting is severely hampered by the inter- and intra-tumoral heterogeneity that propels clonal evolution and adds to antigen variability (27, 28).

**Figure 1:**
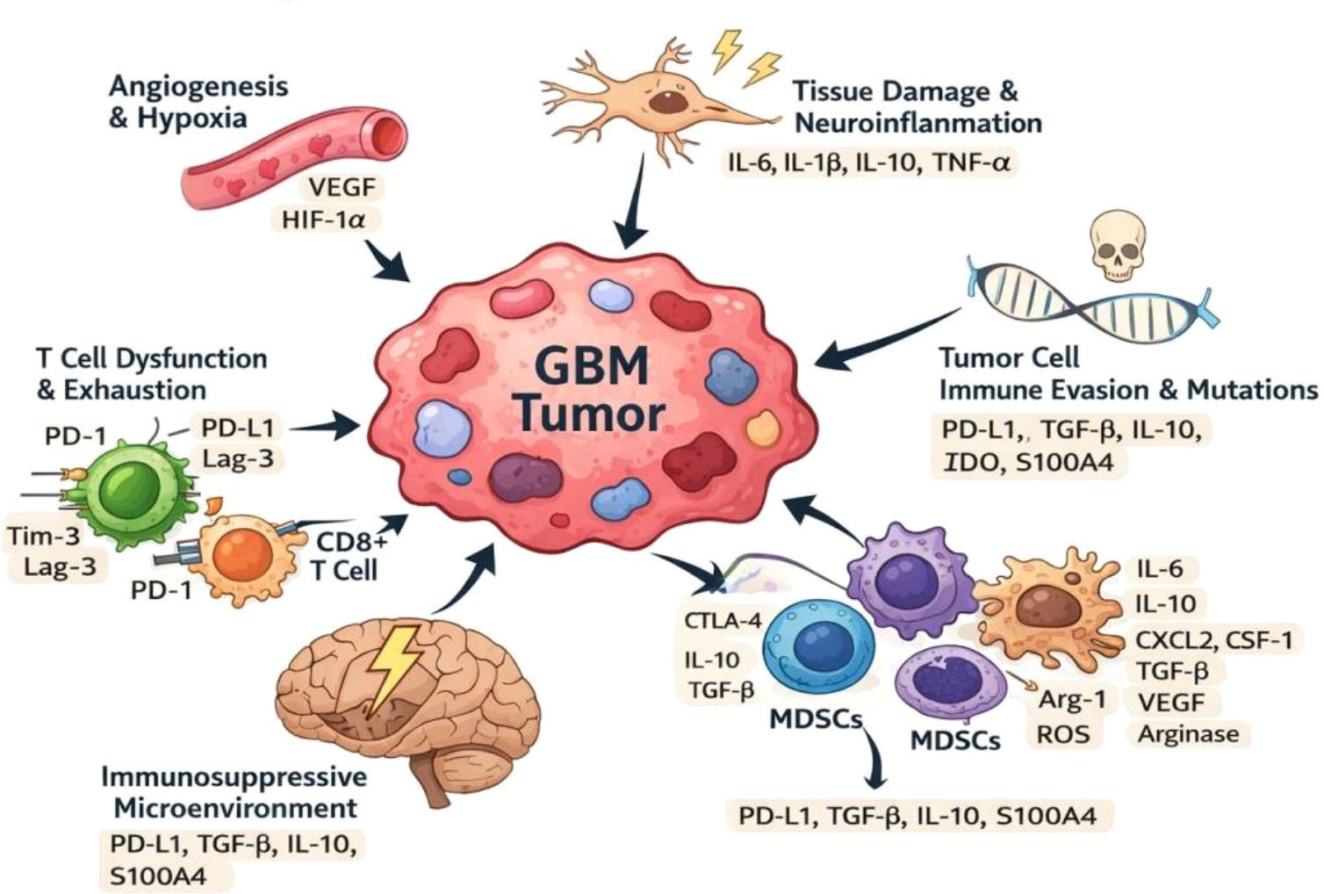
GBM tumor-immune interaction schematic.

Beyond genetic alterations, the immunological landscape of GBM is marked by robust immune evasion mechanisms. The tumor microenvironment is enriched with regulatory T cells (Tregs), tumor-associated macrophages (TAMs), and myeloid-derived suppressor cells (MDSCs), which collectively suppress cytotoxic T lymphocyte activity (24, 25). GBM cells also upregulate immune checkpoint molecules such as PD-L1, leading to T-cell exhaustion and impaired antitumor immunity (25). Furthermore, the secretion of immunosuppressive cytokines including TGF-β and IL-10, dampens immune activation and promotes tumor progression (27). The blood–brain barrier (BBB) and limited lymphatic drainage further restrict immune cell trafficking and antigen presentation within the central nervous system (28). Reasonable vaccine design requires an understanding of this intricate molecular and immunological environment. A framework for finding actionable neoantigens and defeating immune resistance mechanisms in GBM is provided by biocomputational techniques that combine genomic mutations, transcriptomic expression profiles, and immune infiltration signatures (15). **Figure 1** showed schematic representation of GBM tumor-immune interaction.

## Overview of GBM vaccine strategies

Vaccine-based immunotherapy has emerged as a compelling strategy to address the persistent therapeutic challenges associated with Glioblastoma multiforme (GBM) (29). Unlike cytotoxic therapies that aim to directly destroy tumor cells, cancer vaccines are designed to harness and amplify the host immune system’s ability to recognize tumor-specific antigens and generate durable immunologic memory (30, 31). In GBM, where recurrence is nearly universal due to infiltrative growth and residual microscopic disease, vaccines offer the theoretical advantage of long-term immune surveillance (32). Over the past two decades, multiple vaccine platforms, including peptide-based, dendritic cell-based, nucleic acid-based, and personalized neoantigen vaccines have been evaluated in preclinical and clinical settings (33–37).

Clinical results have been inconsistent despite encouraging immunogenicity signals. Consistent therapeutic success has been hampered by the intricacy of GBM biology, which includes significant intra- and inter-tumoral heterogeneity, antigen escape, and immune suppression within the central nervous system (38–40). However, crucial insights from early-phase trials are still being used to guide next-generation vaccination plans, especially when combined with multi-omics and computational techniques.

### Peptide-based vaccines

One of the most researched immunotherapeutic approaches for GBM is peptide-based vaccinations (41). To elicit cytotoxic T lymphocyte (CTL) responses, these vaccines use synthetic peptides that are derived from tumor-associated antigens (TAAs) or tumor-specific mutations. Rindopepimut, which targets the EGFRvIII mutation, an oncogenic deletion variant expressed in a subset of GBM tumors, is one prominent example (42–44). There is a rising optimism for this targeted approach because early-phase trials showed strong antibody and T-cell responses. Survivin, WT1, and mutant IDH1 epitopes are additional peptide targets investigated in GBM (45).

However, larger clinical trials revealed limitations, including lack of significant overall survival benefit in unselected populations (39, 41). One major challenge is antigen heterogeneity and loss, where tumor cells downregulate or eliminate the targeted epitope under immune pressure (39). Furthermore, peptide vaccines are restricted by human leukocyte antigen (HLA) specificity, limiting broad applicability (44). While they are relatively safe, cost-effective, and straightforward to manufacture, their clinical efficacy may require combination with immune checkpoint inhibitors or adjuvants to overcome immune tolerance and exhaustion.

### Dendritic cell vaccines

Dendritic cell vaccines offer a more individualized and immunologically thorough strategy (46). In this approach, autologous dendritic cells are extracted from the patient, ex vivo loaded with tumor lysates, peptides, or RNA, and then reinfused to stimulate tumor-specific T-cell responses (47, 48). DC vaccines may more effectively tackle intratumoral heterogeneity by showcasing a diverse range of tumor antigens, as opposed to single-peptide approaches (49). Numerous clinical studies have indicated extended survival in specific patient subsets receiving DC vaccines, especially when administered post-maximum surgical resection (50–53).

Even though these results are encouraging, developing a DC vaccine is hard because of logistical and biological problems (54). Production is labor-intensive and requires individualized cell processing under strict regulatory conditions, which makes it more expensive and complicated (54). Therapeutic response can also be affected by differences in the quality of dendritic cells, how well they load antigens, and how well a patient’s immune system works (55). The immunosuppressive tumor microenvironment in GBM may also reduce the effectiveness of activated T cells, emphasizing the necessity for combinatorial strategies (56).

### Nucleic acid-based vaccines

Nucleic acid-based vaccines, such as DNA and mRNA platforms, are a quickly growing area of research in GBM immunotherapy (29). These vaccines contain tumor antigens and depend on the host’s cellular machinery to make proteins that trigger an immune response *in vivo* (57). mRNA vaccines are especially appealing because they don’t integrate into the genome, have a good safety record, and can be made quickly (57, 58). They enable the inclusion of multiple epitopes within a single construct, potentially augmenting immune breadth. Additionally, lipid nanoparticles or dendritic cell-targeted platforms can be used to deliver mRNA vaccines in a way that makes antigen presentation better (59, 60). Their flexibility allows for quick changes based on the sequencing data from each person’s tumor. However, there are problems that need to be solved, such as making sure that delivery is good across biological barriers, keeping antigen expression going, and getting around local immune suppression in the brain (58). It is still very important for clinical translation to keep improving delivery systems and adjuvant combinations.

### Personalized neoantigen vaccines

Personalized neoantigen vaccines are designed based on patient-specific somatic mutations identified through next-generation sequencing. Unlike shared tumor-associated antigens, neoantigens arise exclusively in malignant cells, reducing the risk of central immune tolerance and increasing tumor specificity (61). Computational algorithms are used to predict high-affinity MHC-binding epitopes, which are then synthesized into peptide, RNA, or dendritic cell-based vaccine formulations (16).

Early clinical studies show that personalized vaccines can cause strong CD4+ and CD8+ T-cell responses and may help some patients live longer without their disease getting worse. But GBM has a lower mutational burden than other cancers, which makes it hard to find highly immunogenic neoantigens (62, 63). Also, the time and money it takes to make things may make it hard for many people to use them (63). To make personalized GBM vaccine development faster and more accurate, biocomputational pipelines and real-time sequencing technologies will need to work together.

### Biocomputational techniques in vaccine design

The integration of biocomputational techniques has revolutionized vaccine development, particularly in complex malignancies such as Glioblastoma multiforme (GBM) (14). Traditional vaccine discovery relied heavily on experimental screening, which is time-consuming, resource-intensive, and often inefficient in highly heterogeneous tumors (64). In contrast, computational approaches enable systematic mining of genomic, transcriptomic, and proteomic datasets to identify candidate antigens with high immunogenic potential (65). By combining immunoinformatics, structural modeling, systems biology, and artificial intelligence (AI), researchers can design rational, personalized vaccine constructs tailored to tumor-specific molecular profiles (66).

### Reverse vaccinology

Antigen discovery has undergone a paradigm shift thanks to reverse vaccinology (15). This method starts with genomic and proteomic data rather than cultured pathogens or tumor lysates (67). To find overexpressed, mutated, or tumor-specific proteins that could be immunogenic targets in GBM, whole-exome and RNA sequencing datasets are examined (68). To reduce off-target autoimmunity, computational filtering pipelines eliminate proteins that have a high degree of homology to normal tissues (69). The reverse vaccinology workflow typically includes antigen screening, subcellular localization prediction, transmembrane domain analysis, and epitope mapping (70). For GBM, tumor-specific mutations such as EGFRvIII or IDH1 variants can be prioritized using mutation databases and expression profiling (71). This systematic and high-throughput method accelerates candidate selection and reduces experimental burden, making it particularly suitable for personalized vaccine design (72).

### Immunoinformatics approaches

Immunoinformatics tools are central to epitope-based vaccine development. These algorithms predict T-cell and B-cell epitopes based on binding affinity to major histocompatibility complex (MHC) molecules. MHC class I binding predictions are essential for identifying CD8+ cytotoxic T-cell epitopes, whereas MHC class II predictions inform CD4+ helper T-cell responses (73, 74). Advanced algorithms use machine learning models trained on experimentally validated binding datasets to enhance predictive accuracy (75). Computational tools evaluate antigenicity, allergenicity, toxicity, and population coverage in addition to MHC binding (65, 66). For instance, epitope conservancy analysis lowers the risk of antigen escape by ensuring that specific peptides are maintained across tumor subclones (76). In order to maximize vaccine applicability, population coverage analysis assesses the distribution of HLA alleles throughout geographical areas (76). Prior to laboratory validation, these layered analyses work together to improve epitope selection (74).

### Neoantigen identification in GBM

Neoantigen discovery relies on integrating tumor-normal sequencing data to identify nonsynonymous somatic mutations (77, 78). Variant calling pipelines filter raw sequencing reads to detect high-confidence mutations, which are then translated into mutated peptide sequences (79). These candidate neoepitopes undergo computational evaluation for MHC binding affinity and immunogenic potential (79). In GBM, the relatively low tumor mutational burden compared to melanoma or lung cancer presents unique challenges. Therefore, combining DNA sequencing with RNA expression data is critical to ensure that candidate neoantigens are actively transcribed (80). HLA typing algorithms further personalize predictions by tailoring epitope selection to individual patient genotypes (81). This precision-driven approach enhances the likelihood of generating clinically meaningful immune responses (82).

### Structural bioinformatics and molecular modeling

Structural bioinformatics strengthens vaccine design by evaluating the three-dimensional interactions between epitopes and immune receptors (83). Molecular docking simulations model peptide-MHC binding conformations, estimating binding stability and interaction energies (84).These simulations help validate immunoinformatics predictions and identify structurally favorable epitopes (85). By evaluating complex stability over time under physiological conditions, molecular dynamics simulations offer further insight (82, 84). When experimental structures are not available, protein structure prediction tools, such as deep learning-based platforms, enable modeling of mutated tumor antigens (86). In multi-epitope constructs, structural validation can direct linker selection and guarantee optimal epitope presentation (83). The logical design of stable and immunogenic vaccine candidates is enhanced by this structural improvement. Immune-related signaling cascades linked to particular antigens are further revealed by pathway enrichment analyses (85). Prioritizing targets expressed in immunologically active tumor regions is made easier by integrating immune infiltration data from transcriptomic deconvolution algorithms (87). Systems biology frameworks facilitate the selection of therapeutically actionable and biologically relevant vaccine targets by charting the interactions between immune components and tumor cells (82, 84).

### Systems biology and network analysis

Systems biology approaches contextualize candidate antigens within broader cellular networks (88). Protein-protein interaction (PPI) networks identify hub genes and critical signaling nodes involved in GBM pathogenesis (89). Targeting central network components may enhance therapeutic impact by disrupting multiple oncogenic pathways simultaneously (90). Pathway enrichment analyses elucidate immune-related signaling cascades linked to specific antigens. Combining immune infiltration data from transcriptomic deconvolution algorithms helps to prioritize targets that are expressed in tumor areas that are immunologically active (90, 91). Systems biology frameworks facilitate the identification of biologically pertinent and therapeutically viable vaccine targets by elucidating interactions between tumor cells and immune components (92).

### Machine learning and artificial intelligence

**Figure 2:**
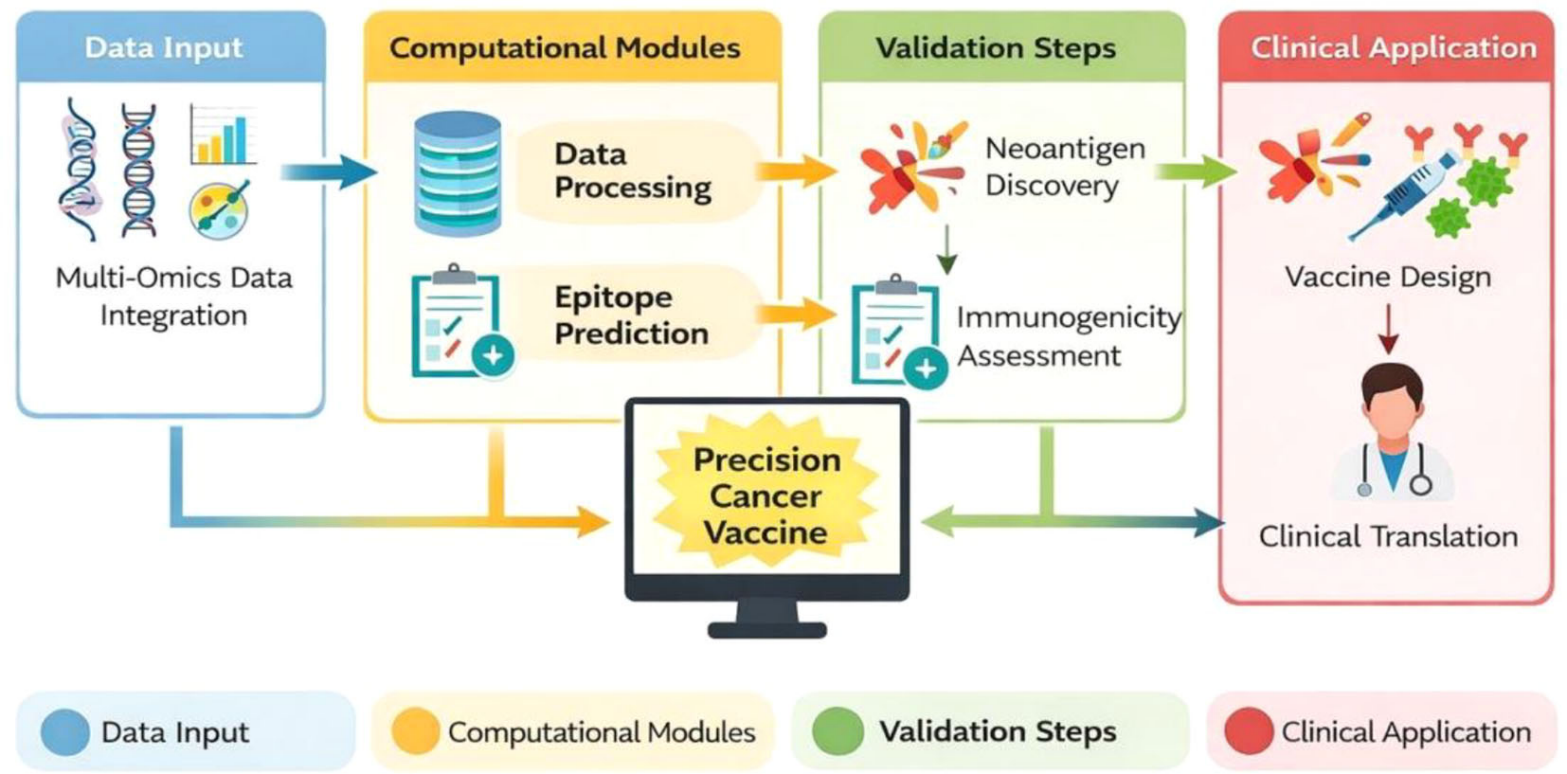
Workflow of computational vaccine development pipeline.

Machine learning and AI technologies are increasingly applied to optimize vaccine design (93). Supervised learning models predict epitope immunogenicity based on sequence features, binding affinity, and structural parameters (94). Deep learning algorithms improve accuracy in MHC binding prediction and neoantigen prioritization by identifying nonlinear patterns within large datasets (95). AI-driven approaches also facilitate integration of multi-omics data, including genomics, transcriptomics, proteomics, and single-cell sequencing (96). Predictive models can estimate patient-specific vaccine response probabilities, enabling risk stratification and precision immunotherapy planning (97). Furthermore, reinforcement learning frameworks are being explored to iteratively optimize vaccine constructs based on simulated immune response feedback. **Figure 2** showed the schematic pipeline of computational vaccine development.

## Multi-epitope vaccine design framework

The development of multi-epitope vaccines represents a rational and highly targeted strategy for immunotherapy in Glioblastoma multiforme (GBM) (98). Unlike single-antigen vaccines, multi-epitope constructs integrate multiple immunogenic regions derived from tumor-associated antigens and neoantigens into a single platform (99). This approach enhances immune coverage, addresses tumor heterogeneity, and reduces the likelihood of immune escape (99). Biocomputational tools play a central role in designing these vaccines, enabling precise selection, optimization, and validation of epitopes prior to experimental and clinical evaluation (13).

### Epitope selection and prioritization

The precise identification of immunogenic epitopes is the cornerstone of multi-epitope vaccine design (100). MHC class I and class II binding peptides are predicted by computational pipelines, which guarantee the activation of CD8+ cytotoxic T lymphocytes and CD4+ helper T cells, respectively (101). To prevent autoimmune reactions, specific epitopes are further screened for high antigenicity, low allergenicity, non-toxicity, and minimal homology with human self-proteins (102). To maximize tumor specificity in GBM, epitopes derived from overexpressed oncogenic proteins or tumor-specific mutations are given priority (103). Population coverage analysis is also incorporated to ensure that selected epitopes are relevant across diverse human leukocyte antigen (HLA) alleles (99). This is particularly important for developing broadly applicable vaccines or optimizing personalized constructs (101). Additionally, epitope conservancy across tumor subclones is evaluated to minimize the risk of antigen loss during tumor evolution (100).

### Vaccine construct design

High-quality epitopes are assembled into a single chimeric construct after they have been chosen. In order to maintain the structural integrity of individual epitopes and enable appropriate processing and presentation by antigen-presenting cells, short peptide linkers are strategically employed (104). Linkers that are frequently used, like AAY or GPGPG sequences, decrease junctional immunogenicity and increase epitope cleavage (105). To improve immune activation, adjuvants are added to the construct. These could be carrier proteins that increase antigen presentation and immune recognition, cytokine sequences, or toll-like receptor (TLR) agonists (106). To guarantee effective expression and delivery, physicochemical characteristics like stability, solubility, and molecular weight are also taken into account during the design process (105).

### Structural modeling and validation

The three-dimensional conformation of the vaccine construct is modeled using structural bioinformatics tools (107). This stage guarantees that the overall structure is stable under physiological conditions and that epitopes are still available for immune recognition (108). Molecular docking studies assess how epitopes interact with MHC molecules or toll-like receptors, offering information about binding affinity and immunogenic potential (109). By evaluating the construct’s stability over time in a biologically simulated environment, molecular dynamics simulations help to further refine it (110). By identifying possible structural flaws and directing optimization prior to experimental validation, these analyses lower failure rates in subsequent research (111).

### Codon optimization and in silico cloning

Codon optimization is used for nucleic acid-based vaccine platforms in order to improve gene expression in the selected host system, such as bacterial or human expression systems (111, 112). This entails increasing translational efficiency and protein yield by modifying codon usage to suit host preferences (113). GC content and mRNA stability are two more parameters that are optimized (114). By simulating the insertion of the optimized gene sequence into expression vectors, in silico cloning techniques guarantee compatibility with regulatory elements and restriction sites (115). This stage expedites the transition from computational design to experimental production and simplifies laboratory workflows (107). Emerging in silico modeling frameworks offer a powerful means of understanding and optimizing interactions between vaccine-induced immune responses and the suppressive tumor microenvironment in Glioblastoma multiforme (GBM) (111). Agent-based models (ABMs) enable simulation of individual cellular behaviors, such as T-cell activation, migration, and exhaustion, within a spatially heterogeneous tumor landscape, capturing interactions with regulatory T cells, tumor-associated macrophages, and myeloid-derived suppressor cells (113). Complementarily, systems biology models use differential equations and network-based approaches to represent signaling pathways (e.g., PD-1/PD-L1, TGF-β) that regulate immune suppression and tumor progression (114, 115). These computational tools allow researchers to test hypothetical vaccine strategies, predict immune dynamics under different therapeutic combinations, and identify conditions that favor effective antitumor immunity (115). Importantly, such models can incorporate patient-specific data, enabling personalized simulations that guide vaccine design, dosing strategies, and combination therapies prior to clinical validation, thereby improving translational efficiency (115).

### Immune simulation and validation

The host immune response to the intended vaccine is predicted using in silico immune simulation platforms (113). Following vaccination, these models calculate the production of antibodies, T-cell activation, cytokine release, and immunological memory formation (109, 116). Researchers can predict long-term efficacy and optimize vaccination schedules by modeling various dosing regimens (104, 117). In order to create highly customized and possibly successful GBM vaccines, the multi-epitope vaccine design framework combines immunoinformatics, structural biology, and systems modeling. This computationally driven method improves accuracy, shortens the time needed for development, and raises the possibility of a successful clinical translation (98, 99, 109).

## Integration with multi-omics data

A key component of developing a precision vaccine for glioblastoma multiforme (GBM) is the integration of multi-omics data (118). Single-layer analyses frequently fall short of capturing the intricacy of tumor biology due to the significant inter- and intra-tumoral heterogeneity that characterizes GBM (119). A thorough characterization of tumor-specific changes and immune interactions is made possible by multi-omics approaches, which include genomics, transcriptomics, proteomics, epigenomics, and emerging spatial technologies (120). Researchers can find high-confidence vaccine targets and create customized immunotherapeutic approaches with increased efficacy and specificity by merging these datasets (121).

### Genomics and transcriptomics

Genomic analysis, particularly through whole-exome sequencing (WES), provides critical insight into somatic mutations that give rise to tumor-specific neoantigens (122). In GBM, identifying nonsynonymous mutations allows for the prediction of candidate epitopes that are uniquely expressed in tumor cells (122, 123). However, not all mutations are biologically relevant or expressed at sufficient levels to elicit an immune response (123). Therefore, integrating transcriptomic data from RNA sequencing (RNA-seq) is essential to confirm gene expression and prioritize functionally active targets (124). Transcriptomics also helps find genes that are expressed differently and immune-related signatures in the tumor microenvironment (122). Gene expression profiling can show that immune checkpoint molecules, cytokines, and pathways that help the immune system avoid detection are all turned up (125). These data guide vaccine target selection and potential combination strategies, including the pairing of vaccines with immune checkpoint inhibitors (126).

### Proteomics and epigenomics

Proteomic analysis provides an additional layer of validation by verifying the actual existence and quantity of proteins originating from candidate genes (127). Proteomics helps narrow down the list of vaccine targets found through genomic and transcriptomic analyses because protein expression ultimately decides how available an antigen is for immune recognition (128, 129). Mass spectrometry and other methods can also find peptides that are naturally present on major histocompatibility complex (MHC) molecules (130). This gives direct proof of immunogenic epitopes. Epigenomic profiling, encompassing DNA methylation and histone modification analyses, elucidates gene regulation in GBM (131). Epigenetic changes can affect how antigens are expressed, how tumors avoid the immune system, and how tumors grow (132). For instance, methylation patterns might turn off tumor suppressor genes or change the way antigens are presented (128). Adding epigenomic data helps find regulatory mechanisms that could affect how well a vaccine works and shows new therapeutic targets (130).

### Single-cell and spatial omics

Single-cell sequencing technologies have revolutionized the comprehension of cellular heterogeneity in GBM (133). Single-cell RNA sequencing (scRNA-seq) can find different types of cells in a tumor or immune system by looking at each one separately (95, 134, 135). These include cancer stem-like cells, immune infiltrates, and stromal components (133). This level of detail makes it possible to find antigens and immune escape mechanisms that are specific to certain cell types, which may not be clear in bulk analyses (136). Spatial transcriptomics improves this understanding even more by keeping the cells in the tumor microenvironment in their proper places (136). With this method, scientists can see how immune cells, antigen expression, and signaling pathways are spread out across different parts of a tumor (95, 135). These kinds of spatial insights are very important for making vaccines that work well against tumor niches that are important for the immune system and that can overcome localized immune suppression (137).

### Toward personalized vaccine pipelines

The integration of multi-omics data supports development of fully personalized vaccine pipelines for GBM (128). Ultimately, multi-omics integration allows for a transition from broad vaccine approaches to targeted immunotherapy (63). There are still some problems to solve, such as the complexity of the data, the need for more computing power, and the need for treatments that can be used in more than one clinic (138). However, this method has a lot of potential to make vaccine-based treatments for GBM more effective.

## Comparatively critique of computational vaccine development pipelines

Computational vaccine development pipelines are becoming an important part of designing immunotherapy for Glioblastoma multiforme (GBM) (124–128). Antigen discovery, epitope prediction, structural validation, and immune response modeling are all parts of these pipelines (86, 109, 111). They have many benefits over traditional experimental methods, but their performance depends on the method used, the quality of the data, and how well they work in the clinic (120). It is therefore necessary to do a critical comparison of the most important parts of the pipeline. **Table 1** showed a comparative analysis of different computational vaccine development pipelines.

**Table 1:** Comparative analysis of vaccine platforms in glioblastoma multiforme.

Parameter	Peptide vaccines	Dendritic cell (DC) vaccines	mRNA vaccines	Neoantigen vaccines
Mechanism	Synthetic tumor-associated peptides	Autologous DCs loaded with antigens	mRNA encoding tumor antigens	Patient-specific mutated antigens
Efficacy	Moderate	Variable	Promising	High (theoretical)
Specificity	Low–moderate	Moderate–high	High	Very high
Scalability	High	Low	High	Low–moderate
Cost	Low	High	Moderate	Very high
Timeline	Short	Long	Short–moderate	Long
Feasibility	High	Moderate	High	Limited
Advantages	Simple, cost-effective	Broad antigen presentation	Rapid, flexible	Highly personalized
Limitations	Antigen escape	Expensive, complex	Delivery challenges	Cost, time
Examples	Rindopepimut	DCVax-L	mRNA platforms	Personalized trials

### Reverse vaccinology vs. neoantigen-based pipelines

Reverse vaccinology pipelines depend on screening tumor-associated antigens (TAAs) obtained from genomic and proteomic datasets (15, 67). Their main strengths are that they can be scaled up and are fast, which makes it easy to find conserved targets across different groups of patients (66). But they are limited by central immune tolerance because many TAAs are not completely tumor-specific (14, 65). This makes them less likely to cause immune reactions and more likely to cause side effects (64).

Neoantigen-based pipelines, on the other hand, focus on somatic mutations that are unique to each patient (76). This makes them very specific and less tolerant of the immune system (79). These pipelines are great for personalized medicine because they make targets that are only found in tumors (74, 76). But they are limited by the low tumor mutational burden in GBM, the high computational demand, and the long turnaround times, which could slow down their use in the clinic (75).

### Immunoinformatics tools vs. experimental validation

Immunoinformatics-based pipelines, such as MHC binding prediction and antigenicity scoring, make it possible to screen a lot of epitopes quickly and cheaply (109, 110). Tools that use machine learning have made predictions more accurate, especially when it comes to peptide-MHC interactions (95, 139). However, these tools frequently yield false positives, as predicted epitopes may not undergo natural processing or presentation *in vivo* (109).

Experimental validation pipelines, like immunopeptidomic based on mass spectrometry, give direct proof of antigen presentation, which makes biological relevance much better (113). But they take a lot of time, money, and resources, and they don’t work well in large groups, which makes it hard to use them in quick vaccine development.(12 The best strategy is often a mix of computational screening and targeted experimental validation.

### Structural bioinformatics vs. sequence-based prediction

Sequence-based pipelines put epitope selection first by using linear peptide features and predictions of binding affinity (133). These methods are fast and can be used in many situations, but they might not take into account the conformational and structural factors that affect immunogenicity (134, 136).

Structural bioinformatics pipelines use molecular docking and molecular dynamics simulations to look at how three-dimensional epitopes interact with MHCs, which makes predictions more accurate (135). These methods make things more accurate, but they need a lot of computer power and aren’t as practical for screening on a large scale (137). So, structural validation is usually used in the later stages of pipeline refinement (95).

### Machine learning/AI-driven pipelines vs. rule-based models

Traditional rule-based pipelines depend on preset thresholds, like binding affinity cutoffs, to be clear and repeatable (95). But they aren’t very flexible and might not be able to capture complicated biological interactions (96).

AI-driven pipelines use deep learning to find nonlinear patterns in big datasets, which makes it easier to predict how well a vaccine will work and how well it will work (93). Even with these benefits, they are limited by dataset bias, overfitting, and a lack of external validation, which could make them less useful for generalizing, especially in groups that aren’t well represented (94).

### Integrated multi-omics pipelines

Multi-omics pipelines use genomic, transcriptomic, and proteomic data to find the most important biological targets (102). These methods provide thorough tumor profiling and better target selection (103). But they are limited by data heterogeneity, problems with integration, and high computational costs, which can make it hard to use them in a lot of clinical settings (100).

## Challenges in computational GBM vaccine development

Despite significant advances in biocomputational approaches, several challenges continue to limit the successful translation of vaccine strategies for Glioblastoma multiforme (GBM) (140). These challenges arise from the biological complexity of the disease, limitations of current computational tools, and barriers to clinical implementation. Addressing these issues is critical to realizing the full potential of precision vaccine design in neuro-oncology (140).

### Tumor heterogeneity and antigen variability

A major challenge in GBM vaccine development is extensive inter- and intra-tumoral heterogeneity (141). GBM tumors are made up of many subclonal populations that have different genetic and phenotypic profiles (142). This causes antigen expression to vary (142). Because of this, epitopes found through computational pipelines may only be found in some tumor cells, which lowers the overall effectiveness of the vaccine (143). Tumors can also go through clonal evolution when they are under pressure from treatment, which can cause antigens to be lost or changed (43, 144). Immune editing lets tumor cells hide from the immune system, especially when vaccines only target a small number of epitopes (145). Multi-epitope and personalized vaccine strategies try to solve this problem, but it is still very hard to get an accurate picture of how different tumors are (145).

### Immunosuppressive tumor microenvironment

The GBM tumor microenvironment is profoundly immunosuppressive, limiting the effectiveness of vaccine-induced immune responses (146). High levels of regulatory T cells (Tregs), tumor-associated macrophages (TAMs), and myeloid-derived suppressor cells (MDSCs) inhibit cytotoxic T-cell activity (147, 148). In addition, tumor cells frequently express immune checkpoint molecules such as PD-L1, promoting T-cell exhaustion (149). Even when computationally designed vaccines successfully generate antigen-specific T cells, these cells may fail to infiltrate the tumor or become functionally inactive upon arrival (150). Overcoming this barrier often requires combination therapies, such as immune checkpoint inhibitors or modulators of the tumor microenvironment, which adds complexity to treatment design and evaluation (149)4.

Emerging biocomputational approaches provide an opportunity to move beyond descriptive characterization toward actionable targeting of these immunosuppressive networks (151). For instance, systems biology and network-based modeling can be used to map signaling pathways governing TME-mediated immune suppression, enabling identification of key regulatory nodes such as TGF-β and IL-10 (152). These insights can guide the rational selection of vaccine targets that not only stimulate effector T-cell responses but also counteract suppressive signaling (153). Additionally, immunoinformatics pipelines can be adapted to incorporate TME-specific gene expression profiles, prioritizing epitopes that are less susceptible to immune inhibition or that are co-expressed with modulatory pathways (45).

Furthermore, models powered by artificial intelligence can be used to mimic how tumors and immune cells interact in the TME and guess how different combinations of treatments will work. For instance, machine learning algorithms can combine multi-omics and single-cell data to make predictions about how Tregs, TAMs, and MDSCs affect antigen presentation and T-cell activation (154). This information can then be used to create vaccines that work with immune checkpoint inhibitors or TME-modulating agents (153). Computational tools can also help find the best way to add adjuvants or cytokine signals that boost immune activation while keeping down cell populations that stop it. Computational methods can help get around one of the biggest problems in GBM immunotherapy by adding these dynamic and context-specific features to vaccine design pipelines (153). This will change the TME from an immunosuppressive niche to one that supports effective antitumor immunity.

### Blood-brain barrier and immune accessibility

The blood-brain barrier (BBB) poses a special difficulty for GBM immunotherapy (150). The BBB may limit the effectiveness of vaccines by preventing immune cells and therapeutic agents from entering the central nervous system (155). GBM can cause local BBB disruption, but this disruption is frequently inconsistent and insufficient for reliable immune access (155). Furthermore, due to its restricted lymphatic drainage and antigen presentation, the central nervous system has traditionally been regarded as an immune-privileged site (156). The brain’s immune responses are still strictly controlled, despite new findings that cast doubt on this theory (156). These physiological and anatomical limitations are frequently not fully taken into account by computational models.

Recent progress in computational biology has led to new ways to get around the blood-brain barrier (BBB) problems in Glioblastoma multiforme (GBM) (157). One important strategy is to use machine learning models trained on physicochemical properties like molecular weight, lipophilicity, and hydrogen bonding capacity to predict how permeable the BBB will be in silico (158). These tools help find molecules that are more likely to get into the central nervous system (CNS), which helps scientists design vaccine parts, adjuvants, and delivery systems (159). Also, computational modeling has made nanoparticle-based delivery systems a lot better by fine-tuning things like particle size, surface charge, and ligand functionalization (160, 161). Simulations of receptor-mediated transport mechanisms, such as transferrin or insulin receptors, in conjunction with molecular docking and dynamics studies, augment targeted delivery across the blood-brain barrier (BBB) and enhance tumor-specific localization (161).

Artificial intelligence (AI) enhances these capabilities by simulating immune cell movement across the BBB (162). AI-driven systems can predict how activated T cells get into the CNS and find important modulators like chemokines and adhesion molecules by combining multi-omics data, vascular dynamics, and immune signaling pathways (38). These models also let you test out combination therapies, like vaccines with checkpoint inhibitors or strategies that change the blood-brain barrier (163). Also, computational methods help improve techniques for breaking down the BBB, like focused ultrasound and receptor-targeted carriers (164). Combining spatial transcriptomics and single-cell data makes it possible to find localized BBB breakdowns in tumors, which makes it easier to target vaccine-induced immune responses accurately (165).

### Limitations of computational predictions

Although biocomputational tools have advanced considerably, they still have certain drawbacks (166). Because epitope prediction algorithms rely on pre-existing datasets, they might not fully capture all immunogenicity determinants, such as peptide processing dynamics or post-translational modifications (104). There is still a risk of false positives and false negatives, which calls for thorough experimental validation (104). Furthermore, the majority of computational models may not accurately capture the complexity of *in vivo* immune responses because they are based on oversimplified assumptions (154). There are additional issues with data quality, standardization, and computational cost when integrating various data types, such as proteomics, genomics, and immunological parameters (154).

### Limitations of in silico predictions

Immunoinformatics and machine learning tools have made epitope prediction much better, but they are still not perfect (166). Most algorithms use predicted major histocompatibility complex (MHC) binding affinity as a stand-in for immunogenicity (104). But for immune responses to work, there are many other things that need to happen, such as antigen processing, peptide stability, T-cell receptor recognition, and the overall tumor microenvironment (154).

As a result, many predicted epitopes are false positives, which means that they bind to MHC molecules in silico but are not naturally processed or presented *in vivo* (154). On the other hand, algorithmic limitations may cause potentially immunogenic epitopes to be missed (104). Moreover, contemporary models frequently neglect to integrate post-translational modifications, tumor-specific antigen processing pathways, and dynamic immune interactions (154).

Machine learning based models introduce additional concerns, including dataset bias, overfitting, and lack of external validation (154). Many training datasets are derived from limited populations, reducing generalizability across diverse HLA backgrounds. Human leukocyte antigen (HLA) diversity represents a critical challenge in the global applicability of vaccine strategies for Glioblastoma multiforme (GBM) (154). HLA alleles vary significantly across populations, influencing antigen presentation and the effectiveness of epitope-based vaccines (104). Computational pipelines often rely on datasets derived predominantly from high-income regions, which may not adequately capture HLA variability in underrepresented populations, particularly in Africa and other low-resource settings (63). This limitation can lead to reduced population coverage and diminished vaccine efficacy outside well-studied cohorts. Furthermore, disparities in access to genomic sequencing, bioinformatics infrastructure, and personalized vaccine platforms exacerbate inequities in implementation (155, 156). Addressing these challenges requires incorporating diverse HLA datasets into predictive models, prioritizing population coverage analyses during vaccine design, and developing scalable, cost-effective approaches that can be adapted to resource-limited environments to ensure equitable global benefit (154). These limitations are particularly relevant for global application and precision medicine.

### Failed clinical translation and antigen loss

Immunoinformatics and machine learning tools have made epitope prediction much better, but they are still not perfect. Most algorithms use predicted major histocompatibility complex (MHC) binding affinity as a stand-in for immunogenicity. But for immune responses to work, there are many other things that need to happen, such as antigen processing, peptide stability, T-cell receptor recognition, and the overall tumor microenvironment.

As a result, many predicted epitopes are false positives, which means that they bind to MHC molecules in silico but are not naturally processed or presented *in vivo*. On the other hand, algorithmic limitations may cause potentially immunogenic epitopes to be missed. Moreover, contemporary models frequently neglect to integrate post-translational modifications, tumor-specific antigen processing pathways, and dynamic immune interactions.

### Translational and regulatory barriers

It is still very difficult to close the gap between clinical application and computational design. Rapid sequencing, data analysis, and manufacturing pipelines are necessary for personalized vaccines, which can be costly and time-consuming (167, 168). Approval pathways are uncertain due to the ongoing evolution of regulatory frameworks for customized therapies (167). Large-scale clinical trials are also required to confirm the safety and effectiveness of computationally designed vaccines, but they are challenging to carry out because of patient heterogeneity and small sample sizes (169, 170). Implementation is made more difficult by ethical issues, such as fair access to cutting-edge treatments.

## Clinical translation and ongoing trials

The clinical translation of vaccine-based immunotherapy for Glioblastoma multiforme (GBM) has progressed steadily over the past two decades, with multiple candidates advancing from preclinical development to early- and late-phase clinical trials (171). These studies have primarily evaluated peptide vaccines, dendritic cell (DC) vaccines, and, more recently, personalized neoantigen and nucleic acid-based platforms. While many trials have demonstrated favorable safety profiles and immunogenicity, translating these responses into consistent survival benefits has proven challenging (171, 172). **Table 2** reported summaries of recent trials on vaccine based immunotherapy for glioblastoma multiforme.

**Table 2:** Summary current trials on vaccine based immunotherapy for GBM.

Study	Year	Stage	Category	Patients group	Vaccine type	Primary outcome	Key summary of findings
Hilf et al. (173)	2019	Phase I	Personalized Vaccine	Newly diagnosed GBM	APVAC1 & APVAC2 (Neoepitopes)	Feasibility & Safety	Proved personalized vaccines are feasible and induce specific T-cell responses.
Muragaki et al. (174)	2023	Phase IIb	Autologous Vaccine	Newly diagnosed GBM	Formalin-fixed tumor vaccine	OS & PFS	No overall OS/PFS difference, but benefit seen in total tumor removal cases.
Cho et al. (175)	2012	Phase II	Dendritic Cell Vaccine	Newly diagnosed GBM	Whole-cell lysate DC	Overall Survival	Median OS significantly improved (31.9 vs 15.0 months).
Sakai et al. (176)	2015	Phase I	Dendritic Cell Vaccine	Relapsed Glioma	WT1/Tumor lysate DC	Safety	Safe and immunogenic; 5 patients achieved stable disease.
Erhart et al. (177)	2018	Phase II	Dendritic Cell Vaccine	GBM	Whole tumor lysate DC	OS & PFS	No clinical survival improvement, but strong immune system stimulation (Th1).
Yao et al. (53)	2018	Phase II	Dendritic Cell Vaccine	GBM	GSC-antigen loaded DC	OS & PFS	Prolonged OS in specific molecular subgroups (low B7-H4/IDH1-wt).
Vik-Mo et al. (178)	2013	Clinical Trial	Cancer Stem Cell Vaccine	GBM	CSC mRNA-transfected DC	Safety & PFS	Safe; vaccinated patients had 2.9x longer PFS than controls.
Sampson et al. (179)	2009	Dose Escalation	Dendritic Cell Vaccine	EGFRvIII+ GBM	EGFRvIII-specific DC	Safety	EGFRvIII is a safe, immunogenic target; median OS was 22.8 months.
Mitsuya et al. (51)	2020	Phase II	Dendritic Cell Vaccine	High-grade glioma	Peptide-cocktail DC	Survival	Verified survival-prolonging effect via T-cell activation.
Akiyama et al. (180)	2012	Phase I	Dendritic Cell Vaccine	Recurrent Glioma	5-peptide cocktail DC	Safety	Well-tolerated; one patient showed long-term recurrence-free survival.
Prins et al. (181)	2011	Dose Escalation	Dendritic Cell Vaccine	Newly/Recurrent GBM	Lysate DC + TLR agonists	Safety	Identified mesenchymal gene profiles as a more responsive subgroup.
Ardon et al. (182)	2012	Phase I/II	Dendritic Cell Vaccine	Newly diagnosed GBM	DC-based vaccination	Feasibility & PFS	Integration into standard care is safe; median OS was 18.3 months.
Wang et al. (50)	2020	Safety/Efficacy	Dendritic Cell Vaccine	GBM/Lung Cancer	Personalized TAA mRNA DC	T-cell Response	Induced specific CD4+/CD8+ responses; associated with favorable survival.
Schijns et al. (183)	2015	Phase II	Combined Vaccine	Recurrent GBM	Allo/Auto Glioma Antigens	Safety & OS	Low toxicity; 6-month survival was 100% in vaccine group vs 33% control.
Izumoto et al. (184)	2008	Phase II	Peptide Vaccine	Recurrent WT1+ GBM	Modified 9-mer WT1	Clinical Response	57.1% disease control rate; well tolerated.
Narita et al. (185)	2019	Phase III	Peptide Vaccine	Recurrent GBM	Personalized Peptide (PPV)	Overall Survival	Failed primary OS endpoint, though benefit noted in specific subgroups.
Morita et al. (186)	2006	Phase I/II	Peptide Vaccine	Solid Tumors	Weekly WT1 peptide	Safety	Confirmed acceptable toxicity for weekly schedules.

### Peptide vaccine trials

Among the first vaccines to undergo clinical testing in GBM were those based on peptides (186). The most well-known example is rindopepimut, an EGFRvIII-targeted vaccine that demonstrated promising outcomes in phase II trials, such as strong antibody responses and extended progression-free survival (184). Nevertheless, when compared to standard therapy, the ensuing phase III ACT IV trial was unable to show a statistically significant overall survival benefit (185). The phenomenon of antigen loss, in which tumors downregulated EGFRvIII expression under immune pressure, resulting in therapeutic resistance, was one of the trial’s most important lessons (185). Other peptide vaccines targeting antigens such as WT1 and survivin have also been investigated in early-phase trials (171, 186). These studies have confirmed the ability of peptide vaccines to induce antigen-specific immune responses, but clinical efficacy has been variable (171, 184–186). These findings underscore the limitations of single-antigen targeting and highlight the need for multi-epitope and personalized approaches.

### Dendritic cell vaccine trials

In GBM, dendritic cell vaccines have shown some of the most encouraging clinical outcomes (180). A large phase III trial involving newly diagnosed GBM patients has assessed DCVax-L, an autologous tumor lysate-loaded dendritic cell vaccine (181). In comparison to historical controls, the study found an extension in median overall survival, especially for patients with favorable prognostic factors (51, 53, 177, 179, 182). Crucially, DC vaccines have continuously demonstrated outstanding safety profiles with few serious side effects (178–181). Despite these positive results, trial design flaws, such as the absence of randomized controls in some studies and patient population heterogeneity, have made it difficult to interpret the findings (50, 179, 181). Furthermore, standardization is challenging due to the individualized nature of DC vaccines, which introduces variability in manufacturing and immune response (182). However, translational research in GBM immunotherapy continues to rely heavily on these vaccines.

### Neoantigen and personalized vaccine trials

Recent advances in next-generation sequencing and immunoinformatics have enabled the development of personalized neoantigen vaccines, which are now being evaluated in early-phase clinical trial (173). These vaccines are tailored to individual patients based on tumor-specific mutations and HLA profiles (173). Preliminary studies have demonstrated the feasibility of this approach, with evidence of robust CD4+ and CD8+ T-cell responses against selected neoantigens (79, 80). Although clinical data remain limited, early results suggest that personalized vaccines may improve immune specificity and reduce off-target effects (173). However, challenges such as long production timelines, high costs, and the relatively low mutational burden of GBM continue to limit widespread application (187). Ongoing trials are exploring strategies to streamline vaccine production and enhance immunogenicity (188).

### Combination strategies and emerging approaches

Current clinical research is increasingly concentrated on combination approaches due to the limitations of monotherapy (189). To improve antitumor responses and overcome T-cell exhaustion, vaccines are being tested in conjunction with immune checkpoint inhibitors, such as PD-1/PD-L1 blockers (189). Other combinations include oncolytic viruses, which can promote local immune activation, and radiotherapy, which may enhance antigen release and presentation (189).

Clinical evaluation is also being conducted on emerging platforms such as nanoparticle delivery systems and mRNA-based vaccines (59, 190). These technologies have the potential for multi-epitope targeting, increased flexibility, and quick customization (59). Optimizing results in clinical trials will depend on the integration of biocomputational design with adaptive trial frameworks.

## Future directions

The future of vaccine development for Glioblastoma multiforme (GBM) lies at the intersection of computational innovation, precision medicine, and combinatorial immunotherapy. While current approaches have demonstrated safety and immunogenic potential, achieving consistent and durable clinical benefit will require more adaptive, individualized, and biologically informed strategies (50, 51, 53, 171, 172, 177–189). Advances in artificial intelligence (AI), multi-omics integration, and next-generation delivery platforms are expected to play a transformative role in overcoming existing limitations (66, 93, 107, 132). The use of AI-driven precision immunotherapy is one of the most promising direction (132). In order to predict highly immunogenic neoantigens and predict patient-specific vaccination responses, machine learning models can incorporate genomic, transcriptomic, proteomic, and clinical data (132). Additionally, by identifying the best epitope combinations, these predictive systems can lower the chance of immune escape and increase the effectiveness of vaccines (93, 107, 132). AI can also help create “digital twin” models, which are computational replicas of specific patients that mimic tumor-immune interactions and enable in silico testing of vaccine strategies prior to clinical implementation (132).

Another critical area is the integration of vaccine therapies with other immunomodulatory treatments. Combining vaccines with immune checkpoint inhibitors, such as PD-1/PD-L1 or CTLA-4 blockers, may enhance T-cell activation and overcome tumor-induced immune suppression (189). Similarly, radiotherapy and certain chemotherapeutic agents can promote immunogenic cell death, increasing antigen availability and improving vaccine responsiveness (169). The incorporation of oncolytic viruses and chimeric antigen receptor T-cell (CAR-T) therapies further expands the therapeutic landscape, offering synergistic mechanisms to target GBM (189). Future paths are also being shaped by developments in vaccine platforms. Rapid, scalable, and customizable vaccine production is made possible by viral vectors, nanoparticle delivery systems, and mRNA-based vaccines (59, 190). Because they enable real-time adaptation to tumor evolution, these technologies are especially well-suited for customized neoantigen vaccines (173). Therapeutic efficacy will be further increased by advancements in delivery strategies targeted at improving penetration across the blood–brain barrier (150, 155).

To enhance the rational design of combination therapies for Glioblastoma multiforme (GBM), computational approaches can be leveraged to systematically identify synergistic partners for vaccine-based immunotherapy. Multi-omics integration, particularly transcriptomic and single-cell RNA sequencing data, enables the characterization of immune states such as T-cell exhaustion and macrophage polarization within the tumor microenvironment (118). For instance, elevated expression of exhaustion markers (e.g., PD-1, LAG-3) or enrichment of M2-like tumor-associated macrophages can be used to stratify patients who may benefit from combining vaccines with immune checkpoint inhibitors or macrophage-reprogramming agents (119). Systems biology frameworks further allow the mapping of signaling networks that drive immune suppression, facilitating the identification of co-targetable pathways such as TGF-β or IL-10 signaling (121). These data-driven strategies support the development of personalized, mechanism-based combination therapies rather than empirical treatment selection.

In parallel, spatially resolved and dynamic computational modeling approaches offer critical insights into how vaccines interact with the heterogeneous GBM microenvironment (125). Spatial transcriptomics can distinguish immunologically “hot” regions with active immune infiltration from “cold” regions characterized by immune exclusion, thereby informing both antigen targeting and optimal delivery strategies, such as intratumoral versus systemic administration (122, 123). Moreover, emerging in silico frameworks, including agent-based models and systems-level simulations, enable the dynamic modeling of interactions between vaccine-induced T cells and suppressive elements such as Tregs, TAMs, and MDSCs (152, 153). These models can predict how immune cells traffic, expand, and function within the tumor niche under different therapeutic conditions, providing a platform to test and optimize vaccine strategies prior to clinical translation (153). Collectively, integrating multi-omics, spatial data, and computational simulations offers a powerful, forward-looking approach to overcoming TME-associated resistance in GBM.

To strengthen future research directions in Glioblastoma multiforme (GBM), there is a need to move beyond conceptual frameworks toward actionable, technology-driven strategies. One promising priority is the development of lipid nanoparticle (LNP)-based mRNA vaccine platforms, which enable rapid, scalable, and personalized antigen delivery while improving stability and cellular uptake (191, 192). Parallel efforts should focus on blood-brain barrier (BBB) penetrating delivery systems, including ligand-targeted nanoparticles and receptor-mediated transport models, guided by computational optimization to enhance CNS-specific immune activation (193). Additionally, future studies should adopt adaptive clinical trial designs, such as basket and platform trials, allowing real-time modification of treatment arms based on biomarker responses and patient stratification. Integrating longitudinal biomarkers such as circulating tumor DNA, immune profiling, and imaging signatures, into these trial frameworks will facilitate dynamic assessment of vaccine efficacy and enable precision-guided therapeutic adjustments. Collectively, these approaches provide concrete, testable pathways to accelerate translation of computational vaccine strategies into clinically meaningful outcomes.

Lastly, real-time biomarker monitoring and flexible clinical trial designs will be crucial for quickening translation. Longitudinal imaging, circulating tumor DNA analysis, and immune profiling can all be used to provide early response indicators and direct treatment changes (7, 31, 32). Combining computational accuracy, biological understanding, and clinical flexibility to develop highly customized and successful immunotherapeutic approaches will be critical to the development of GBM vaccines in the future (142, 187).

## Conclusion

Glioblastoma multiforme (GBM) remains one of the most formidable challenges in neuro-oncology, characterized by aggressive behavior, profound heterogeneity, and resistance to conventional therapies. Despite decades of research, current treatment modalities including surgical resection, radiotherapy, and chemotherapy, provide only limited survival benefit, with recurrence being almost inevitable. In this context, vaccine-based immunotherapy has emerged as a promising strategy aimed at eliciting durable and tumor-specific immune responses capable of targeting residual and recurrent disease. This review emphasizes the revolutionary impact of biocomputational techniques in enhancing GBM vaccine development. Methods like reverse vaccinology, immunoinformatics, structural bioinformatics, systems biology, and artificial intelligence have made it possible to quickly find and rank tumor antigens and neoantigens. These tools make it easier to design multi-epitope and personalized vaccines in a logical way. They also fix some of the biggest problems with traditional vaccine strategies, like antigen heterogeneity and immune escape. Moreover, the amalgamation of multi-omics data encompassing genomics, transcriptomics, proteomics, and single-cell analyses has augmented the accuracy and biological significance of prospective vaccine targets.

However, significant challenges remain. The immunosuppressive tumor microenvironment, variability in antigen expression, limitations of computational predictions, and barriers to clinical translation continue to hinder consistent therapeutic success. Clinical trials to date have demonstrated encouraging safety profiles and immunogenicity but have yielded variable survival outcomes. These findings underscore the need for more refined, adaptive, and combination-based approaches. In the future, the combination of computational innovation, personalized medicine, and advanced immunotherapy platforms will be a powerful way to improve outcomes in GBM. To make vaccines work better and get them into clinical practice faster, it will be important to use artificial intelligence, real-time biomarker monitoring, and flexible clinical trial designs. In the end, a multidisciplinary and integrative approach will be necessary to fully realize the potential of vaccine-based therapies and advance toward significant, long-term management of GBM.
